# Miscarriage on Endometriosis and Adenomyosis in Women by Assisted Reproductive Technology or with Spontaneous Conception: A Systematic Review and Meta-Analysis

**DOI:** 10.1155/2020/4381346

**Published:** 2020-12-08

**Authors:** Yangxue Huang, Xianhong Zhao, Yiyuan Chen, Jie Wang, Weilin Zheng, Lixing Cao

**Affiliations:** ^1^Guangzhou University of Chinese Medicine, Guangzhou, Guangdong Province, China; ^2^Gynecology, The Second Affiliated Hospital of Guangzhou University of Chinese Medicine, Guangzhou, Guangdong Province, China

## Abstract

**Background:**

In the past several years, there has been an increasing concern on miscarriage caused by endometriosis or adenomyosis. However, the results reported by different studies remain controversial. The present study is aimed at assessing the impact of endometriosis and adenomyosis on miscarriage.

**Materials and Methods:**

Searches were carried out in PubMed, Embase, and the Cochrane library for studies published from inception until February 29, 2020. The investigators included studies that evaluated miscarriage risk in pregnant women with endometriosis or adenomyosis by assisted reproductive technology (ART), or with spontaneous conception (SC). Miscarriage (<28 weeks) was the primary outcome. The secondary outcomes were antepartum hemorrhage (APH), postpartum hemorrhage (PPH), preterm birth, low birthweight, placenta praevia, placental abruption, ectopic pregnancy, stillbirth, gestational diabetes, preeclampsia, and intrauterine growth restriction (IUGR). Endnote was used for the study collection, and the data analyses were carried out by two authors using Review Manager version 5.2.

**Results:**

Thirty-nine studies, which is comprised of 697,984 women, were included in the present study. Miscarriage risk increased in women with endometriosis in SC (OR: 1.81, 95% CI: 1.44-2.28, *I*^2^ = 96%) compared with those without endometriosis, while women with endometriosis who underwent ART had a similar miscarriage risk, when compared to those with tubal infertility (OR: 1.03, 95% CI: 0.92-1.14, *I*^2^ = 0%). Compared with those without adenomyosis, women with adenomyosis had an augmented miscarriage risk in ART (OR: 2.81, 95% CI: 1.44-5.47, *I*^2^ = 64%). Compared with those without endometriosis, women with endometriosis had higher odds of APH, PPH, preterm birth, stillbirth, and placenta praevia. No difference was observed in the incidence of ectopic pregnancy, placental abruption, pre-eclampsia, gestational diabetes, low birthweight, and IUGR.

**Conclusion:**

Women with endometriosis had an augmented miscarriage risk in SC and a similar miscarriage risk during ART. Adenomyosis was associated with miscarriage in pregnant women using ART.

## 1. Introduction

Endometriosis (EMS) and adenomyosis (AD) are both complicated diseases that have influence on pregnancy outcomes. EMS is identified by the endometrium outside the uterus and is correlated to pelvic pain and infertility [1]. It has been reported that the disease affects up to 10%-15% of women during the reproductive age [2]. Adenomyosis, which is defined as ingrowth of the endometrial tissue into the myometrium [3], is associated with heavy menstrual bleeding and dysmenorrhea. It has been estimated that 20.9% of women are diagnosed with AD through transvaginal sonography (TVS) [4].

In the past several years, there has been an increasing concern on miscarriage caused by EMS or AD. Many studies have assessed the miscarriage risk in women with EMS or AD. However, the results reported from different studies remain controversial, since some studies presented positive results, while other studies reported negative results [5, 6]. Therefore, a systematic review and meta-analysis was carried out to evaluate the impact of EMS or AD on miscarriage in women who are pregnant with spontaneous conception (SC), or by using assisted reproductive technology (ART). The EMS was staged according to the American Fertility Society classification. Where appropriate, EMS I/II was compared with EMS III/IV on miscarriage, and the investigators planned to assess the miscarriage risk according to the types of EMS, including superficial peritoneal endometriosis (SUP), deep infiltrating endometriosis (DIE), and ovarian endometrioma (OMA). Where applicable, the investigators evaluated the effect of EMS or AD on early abortion (at <12 weeks) and late abortion (at ≥12 weeks).

## 2. Materials and Methods

### 2.1. Search Strategy

Electronic databases (PubMed, Embase, and Cochrane library) were searched for published studies from inception to February 29, 2020, in all languages, by two authors, independently. The MeSH terms were as follows: “ademomyosis,”“endometriosis,” “spontaneous abortion,” “miscarriage,” “assisted reproductive technique,” “ovulation induction,” “artificial insemination,” “in vitro fertilization,” “intracytoplasmic sperm injection,” and “embryo transfer.” No restriction for geographic location was applied, and the references were collected by Endnote. In addition, the reference lists of eligible articles and relevant reviews were manually examined to identify potentially available studies. The present meta-analysis was registered with PROSPERO (https://www.crd.york.ac.uk/PROSPERO), and the registration code was CRD42020160594.

### 2.2. Inclusion and Exclusion Criteria

Duplicates were removed prior to the title and abstract screening. The inclusion criteria were as follows: (1) studies that investigated miscarriage risk in pregnant women with SC or using ART; (2) women with EMS or AD who were included in the study group; (3) an appropriate control group; (4) among women with EMS who underwent ART, the control group consisted of only women with tubal infertility; and (5) randomized controlled trials, cohort studies, case control studies, or cross-sectional analysis. The EMS or AD could be preliminarily diagnosed by clinical symptoms, gynecological examination, and instrumental (ultrasound, computed tomography scan, or magnetic resonance imaging) presentation. The golden standard was pathological diagnosis. In addition, the exclusion criteria were as follows: (1) the publication was a conference abstract or a review; (2) the studies were conducted in animals; (3) the outcome did not include miscarriage; and (4) the necessary data was missing. After independently examining the eligibility of studies based on the titles and abstracts, the full texts were reviewed by two authors. A third author was consulted to resolve any discrepancies.

### 2.3. Data Extraction and Quality Assessment

For eligible studies, the data were extracted by two authors independently. A data collection form was designed for the data extraction, which included the first author, publication year, study design, sample size, study location, mode of conception, type of disease, exposure ascertainment, and outcomes. If disagreements appeared, this was discussed with a third reviewer to reach a consensus. If required, the authors of the qualified publications were contacted for detailed results and precise data.

According to the Newcastle-Ottawa Scale (NOS), the investigators evaluated the risk of bias to identify the methodology quality of the eligible studies. Nine items were included in the NOS, which were categorized into three groups: consisted of the selection of the study group and control group (4 scores, indicating selection bias), the comparability of two groups (2 scores, indicating confounding bias), and the identification of either outcome or exposure (3 scores, indicating measurement bias). The outcome assessment of seven or more stars implied a low risk of bias. The risk of methodological bias in the randomized controlled trials (RCTs) was evaluated using the Cochrane risk of bias tool, including randomization, allocation concealment, blinding of participants and researchers, blinding of outcomes assessors, incomplete outcome reporting, selective outcome reporting, and other sources of bias.

### 2.4. Statistical Analysis

The data analyses were independently carried out by two authors using Review Manager version 5.2. If differences occurred, a third author was consulted for evaluation. According to the Cochrane handbook [7], the heterogeneity was measured by *I*^2^. An *I*^2^ value of 0-50% was considered to represent low or moderate heterogeneity, while >50% was taken to indicate substantial heterogeneity. The fixed effects model was applied for the meta-analysis. The random effects model was used when *I*^2^ > 50%. Pregnancy outcomes were depicted using the odds ratio and 95% confidence interval (CI) [8]. *P* < 0.05 was considered statistically significant. Potential publication biases were statistically evaluated using funnel plots and Begg's and Egger's tests [9]. The present study was reported in accordance with the Preferred Reporting Item for Systematic Reviews and Meta-analyses (PRISMA) statement [10].

The primary outcome was miscarriage, which was defined as spontaneous abortion at <28 weeks. The secondary outcomes were preterm birth (defined as birth < 37 gestational weeks), antepartum hemorrhage (APH), postpartum hemorrhage (PPH), low birthweight (defined as birth weight < 2,500 g), placenta praevia (identified by the placenta implanted in the lower uterine segment), placental abruption (defined as partial or complete detachment of the placenta from the myometrium before delivery), ectopic pregnancy, stillbirth, gestational diabetes, preeclampsia, and intrauterine growth restriction (IUGR).

Where applicable, the subgroup analyses for miscarriage risk in women with EMS were performed based on the method of diagnosis (i.e., laparoscopic diagnosis), type of EMS (i.e., ovarian, peritoneal, or deep infiltrating endometriosis), and staging of EMS (I, II, III, or IV). Sensitivity analyses for miscarriage risk were carried out to evaluate the stability and reliability of the pooled results.

## 3. Results

### 3.1. Study Selection

**Figure 1:**
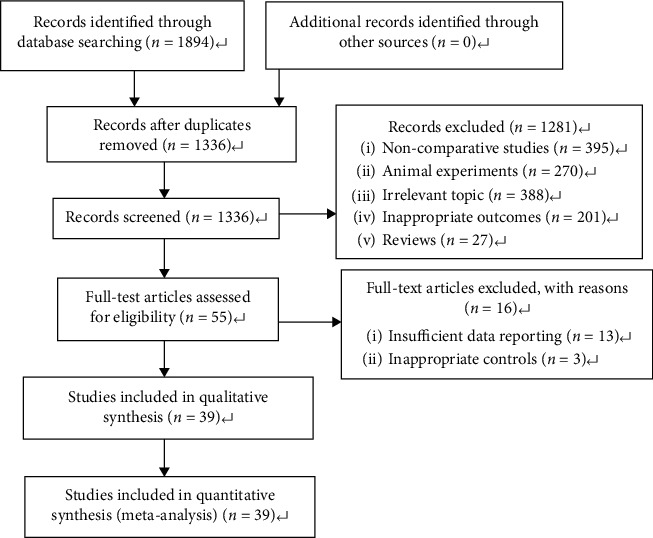
Flow chart of the literature selection.

A total of 1,894 articles were identified using the electronic search strategy. Furthermore, 1,336 articles were evaluated after the duplicates were removed. The eligibility of studies was assessed based on the titles and abstracts, and 1,281 articles were discarded for noncomparative studies (*n* = 395), for animal experiments (*n* = 270), for irrelevant topics (*n* = 388), for inappropriate outcomes (*n* = 201), or for being reviews (*n* = 27). Moreover, 55 articles were eligible for full-text review. Among these, 13 papers were excluded due to inadequate data reporting and 3 studies were excluded because of inappropriate controls. Lastly, 39 publications [11–49], which consisted of 697,984 women, met the present inclusion criteria and were analyzed in the present study (Figure 1).

### 3.2. Characteristics of Eligible Studies

The principal characteristics of the qualified publications are summarized in Table 1. According to the cautious assessment using the NOS, the majority of the studies had scores of 7 or greater (31/38), indicating a low risk of bias. Seven publications had a medium risk of bias, with scores of 6 (Table 2). According to the systematic risk evaluation of methodological bias, the descriptions about allocation concealment and blinding methods were not provided in this RCT (Table 3).

### 3.3. Clinical Outcomes

**Figure 2:**
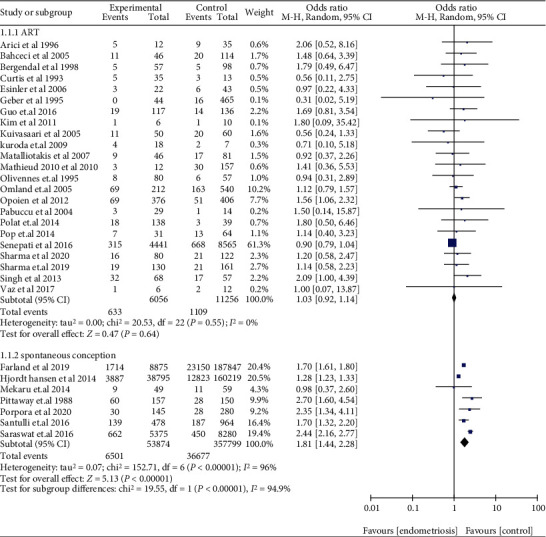
Miscarriage risk in pregnant women with EMS in SC or using ART.

**Figure 3:**
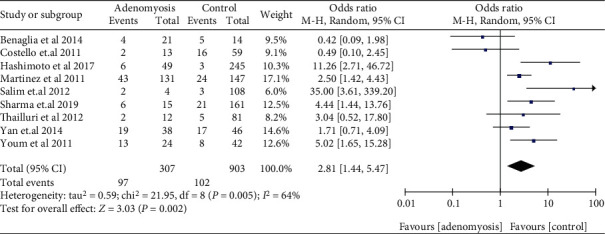
Miscarriage risk in pregnant women with AD in ART.

**Figure 4:**
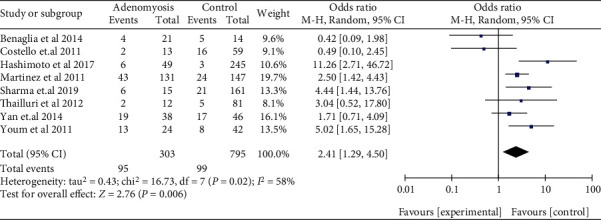
Sensitivity analysis of miscarriage risk in pregnant women with AD.

The risk of miscarriage increased in women with EMS, when compared with those without EMS in SC (OR: 1.81, 95% CI: 1.44-2.28, *I*^2^ = 96%). Among women who underwent ART, women with EMS had a similar miscarriage risk when compared to women with tubal infertility (OR: 1.03, 95% CI: 0.92-1.14, *I*^2^ = 0%) (Figure 2). Compared to women without AD, women who had a prior diagnosis of AD had a higher miscarriage risk in ART (OR: 2.81, 95% CI: 1.44-5.47, *I*^2^ = 64%) (Figure 3). The data of women with AD, who conceived spontaneously, was lacking. In the sensitivity analysis, the results of women with EMS who conceive spontaneously concurred with the pooled results after eliminating anyone study. At the same time, the sensitivity analysis of AD did not alter the conclusion (OR: 2.41, 95% CI: 1.29-4.50, *I*^2^ = 58%) (Figure 4).

**Figure 5:**
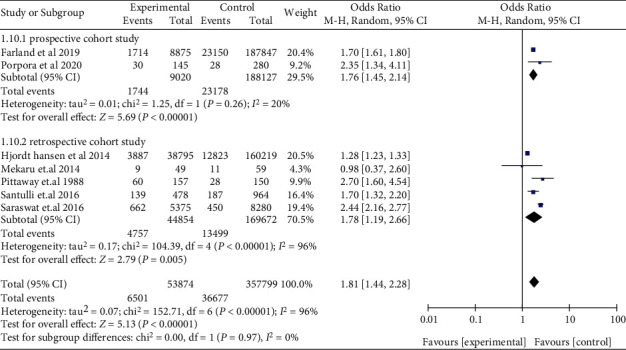
Miscarriage risk in women with EMS in retrospective cohort studies and prospective cohort studies in SC.

**Figure 6:**
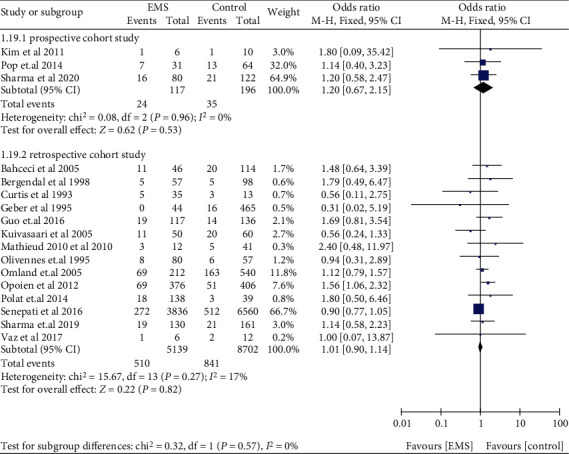
Miscarriage risk in women with EMS in retrospective cohort studies and prospective cohort studies during ART.

**Figure 7:**
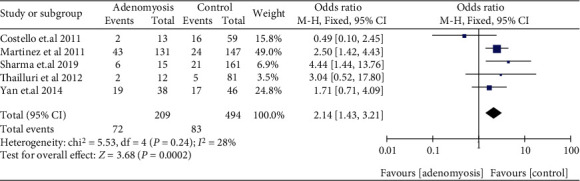
Miscarriage risk in women with AD in retrospective cohort studies during ART.

**Figure 8:**
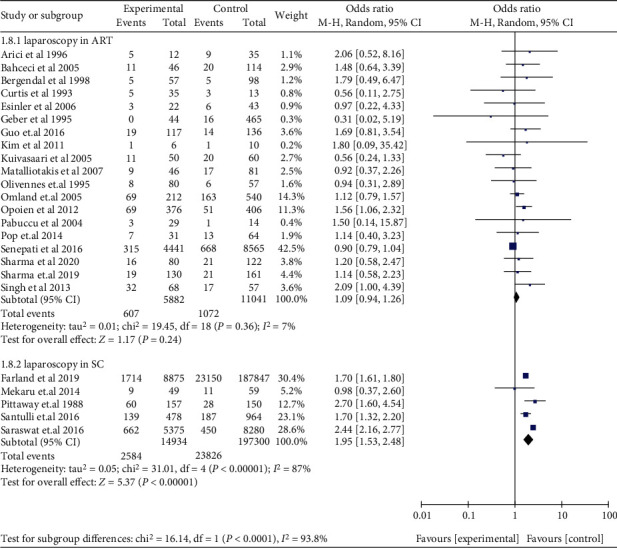
Miscarriage risk in women with EMS diagnosed by laparoscopy.

The subgroup analyses in women with EMS for retrospective cohort studies (OR: 1.78, 95% CI: 1.19-2.66, *I*^2^ = 96%) and prospective cohort studies (OR: 1.76, 95% CI: 1.45-2.14, *I*^2^ = 20%) were consistent with the overall analysis, observing an increased miscarriage risk in SC (Figure 5). Miscarriage risk was similar between women with EMS and tubal infertility who underwent ART in retrospective cohort studies (OR: 1.01, 95% CI: 0.90-1.14, *I*^2^ = 17%), prospective cohort studies (OR: 1.20, 95% CI: 0.67-2.15, *I*^2^ = 0%), and a RCT (OR: 1.50, 5% CI: 0.14-15.87, 1 study) (Figure 6). Women with AD had higher odds of miscarriage in retrospective cohort studies (OR: 2.14, 95% CI: 1.43-3.21, *I*^2^ = 28%) (Figure 7). In the subgroup analysis, the findings of women with EMS diagnosed by laparoscopy remained in line with the overall results, implying an augmented miscarriage risk in women with or without EMS in SC (OR: 1.95, 95% CI: 1.53-2.48, *I*^2^ = 87%) and a similar miscarriage risk between women with EMS and tubal infertility during ART (OR: 1.09, 95% CI: 0.94-1.26, *I*^2^ = 7%) (Figure 8).

**Figure 9:**
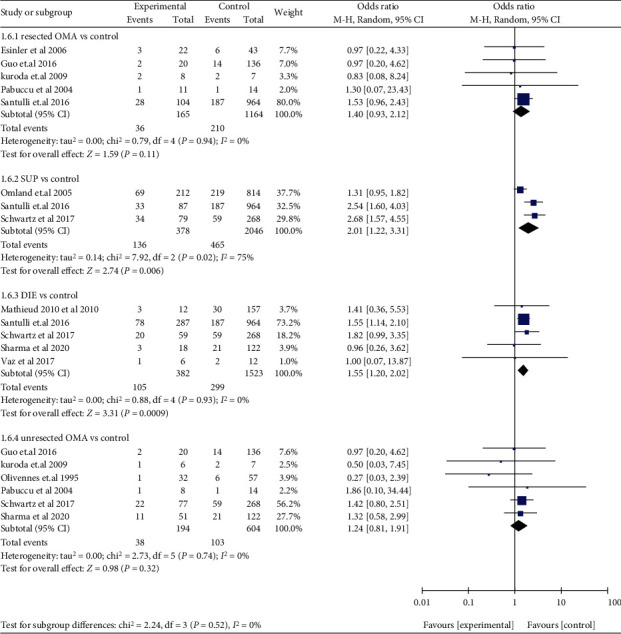
Miscarriage risk in women with resected OMA, unresected OMA, DIE, and SUP.

**Figure 10:**
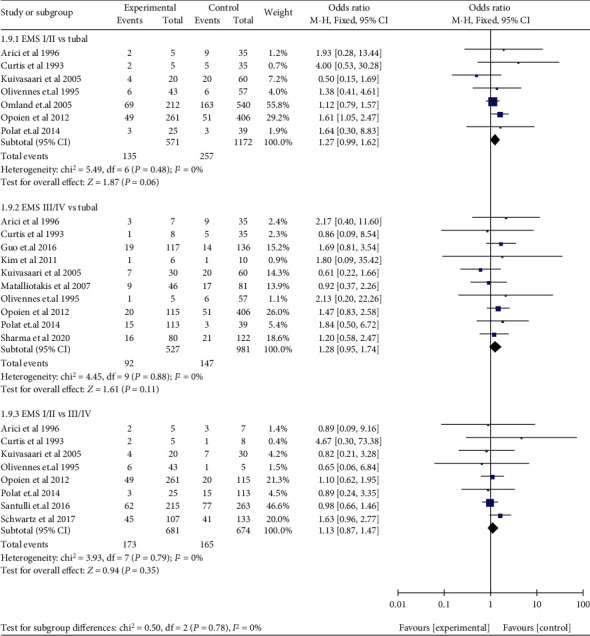
Miscarriage risk in EMS I/II and EMS III/IV.

**Figure 11:**
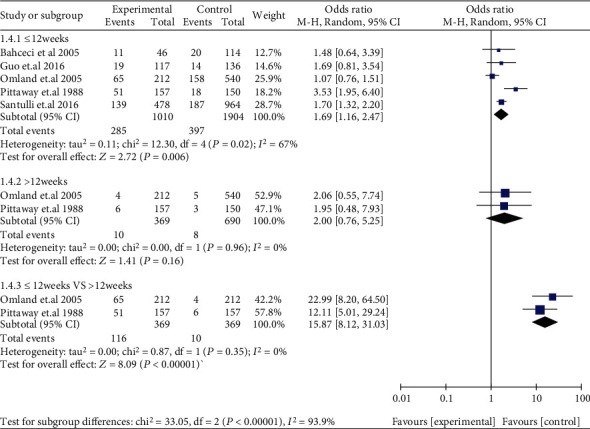
Early abortion and late abortion in women with EMS.

Compared with women without EMS, women with DIE (OR: 1.55, 95% CI: 1.20-2.02, *I*^2^ = 0%) and women with SUP (OR: 2.01, 95% CI: 1.22-3.31, *I*^2^ = 75%) had a higher miscarriage risk, while resected OMA (OR: 1.40, 95% CI: 0.93-2.12, *I*^2^ = 0%) and unresected OMA (OR: 1.24, 95% CI: 0.81-1.91, *I*^2^ = 0%) both had a similar miscarriage risk (Figure 9). Compared with those with tubal infertility, who underwent ART, women with EMS I/II (OR: 1.27, 95% CI: 0.99-1.62, *I*^2^ = 0%) and women with EMS III/IV (OR: 1.28, 95% CI: 0.95-1.74, *I*^2^ = 0%) had a similar miscarriage risk, respectively. Compared with those without EMS, who conceived spontaneously, women with EMS I/II (OR: 1.68, 95% CI: 1.20-2.35, 1 study) and women with EMS III/IV (OR: 1.72, 95% CI: 1.26-2.34, 1 study) had a higher miscarriage risk, respectively. There was no significant difference observed in miscarriage risk when EMS I/II was compared with EMS III/IV (OR: 1.13, 95% CI: 0.87-1.47, *I*^2^ = 0%) (Figure 10). Compared to those without EMS, women with EMS had a higher risk in early abortion (at <12 weeks) (OR: 1.69, 95% CI: 1.16-2.47, *I*^2^ = 67%), while late abortion risk (at ≥12 weeks) (OR: 2.00, 95% CI: 0.76-5.25, *I*^2^ = 0%) was similar in women with or without EMS. In addition, early abortion risk was higher than late abortion risk in women with EMS (OR: 15.87, 95% CI: 8.12-31.03, *I*^2^ = 0%) (Figure 11). A subgroup analysis for early abortion and late abortion in AD was not feasible, because there were insufficient data stratified by week of miscarriage.

**Figure 12:**
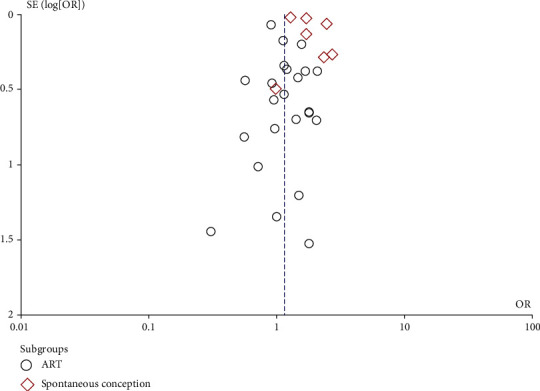
Funnel plot of miscarriage risk in women with EMS.

Since there were less than 10 studies presenting the association between AD and miscarriage, the funnel plot was not conducted for publication bias. Furthermore, the funnel plot was made to describe the miscarriage risk in women with EMS (Figure 12), which was generally in symmetry, with the Begg's test (*P* = 0.301) and Egger's test (*P* = 0.942) implying no publication bias.

**Figure 13:**
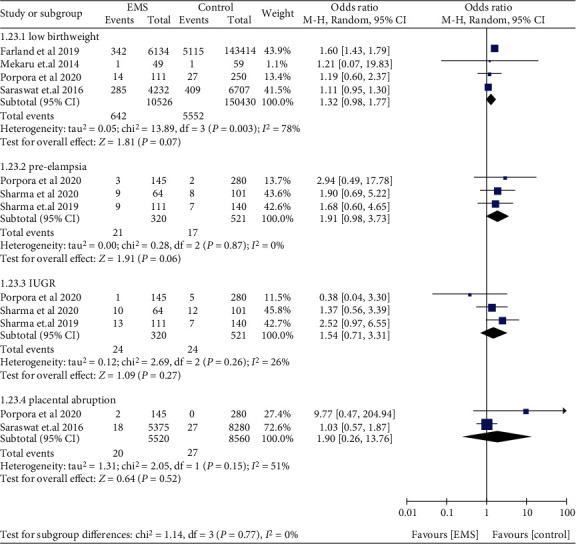
Low birthweight, preeclampsia, IUGR, and placental abruption in women with EMS.

**Figure 14:**
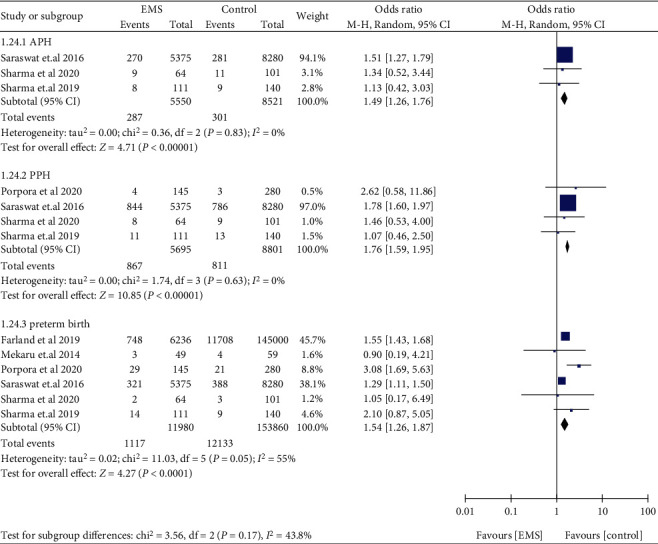
APH, PPH, and preterm birth in women with EMS.

**Figure 15:**
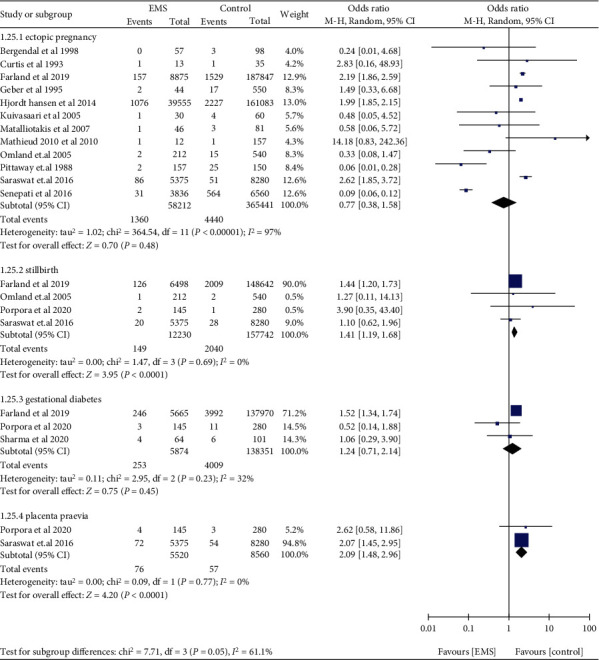
Ectopic pregnancy, stillbirth, gestational diabetes, and placenta praevia in women with EMS.

Women with EMS were not found to be associated with low birthweight (OR: 1.32, 95% CI: 0.98-1.77, *I*^2^ = 78%), placental abruption (OR: 1.90, 95% CI: 0.26-13.76, *I*^2^ = 51%), IUGR (OR: 1.54, 95% CI: 0.71-3.31, *I*^2^ = 26%), and preeclampsia (OR: 1.91, 95% CI: 0.98-3.73, *I*^2^ = 0%) (Figure 13). Compared to those without EMS, women with EMS had higher odds of APH (OR: 1.49, 95% CI: 1.26-1.76, *I*^2^ = 0%), PPH (OR: 1.76, 95% CI: 1.59-1.95, *I*^2^ = 0%), and preterm birth (OR: 1.54, 95% CI: 1.26-1.87, *I*^2^ = 55%) (Figure 14). Women with EMS were more likely to have placenta praevia (OR: 2.09, 95% CI: 1.48-2.96, *I*^2^ = 0%) and stillbirth (OR: 1.41, 95% CI: 1.19-1.68, *I*^2^ = 0%) compared to women without EMS, while no difference was observed in gestational diabetes (OR: 1.24, 95% CI: 0.71-2.14, *I*^2^ = 32%) and ectopic pregnancy (OR: 0.77, 95% CI: 0.38-1.58, *I*^2^ = 97%) (Figure 15).

## 4. Discussion

The present study revealed that EMS is correlated to increased miscarriage risk in pregnant women with SC, while women with EMS had a similar miscarriage risk when compared to those with tubal infertility, who underwent ART. At the same time, an increased miscarriage risk was observed in women with EMS during ART/SC, when compared to those without EMS [50]. No difference was observed in women with or without EMS, who underwent IVF/ICSI [6]. As it is known, EMS was defined as the endometrium outside the uterus, which has major effects on the pelvic environment. The potential explanation might be that EMS generates major effects on the process of fertilization. Therefore, EMS has less impact on women using ART, whose site of fertilization is not in the pelvis. Among women who underwent ART, AD was found to be associated with miscarriage, which is consistent with some literatures [5, 51]. Adenomyosis is identified by ingrowth of the endometrial tissue into the myometrium, which may have a major impact on intrauterine embryos in women using ART.

The sensitivity analyses of miscarriage risk in EMS or AD were both consistent with the whole conclusion, which proves the stability and reliability of the pooled results. In the subgroup analysis, AD was found to be associated with miscarriage in the retrospective cohort study. The findings in the retrospective cohort study, prospective cohort study, and RCT for women with EMS concurred with the overall results, implying the augmented miscarriage risk in women with SC and a similar miscarriage risk in women who underwent ART. Similarly, among women whose EMS was diagnosed by laparoscopy, it was found that there was a similar miscarriage risk in women during ART and an increased miscarriage risk in women who conceived spontaneously.

As it is known, the major indications of ART were various factors of infertility. The risk of spontaneous abortion might be affected by different factors of infertility and not ascribed to EMS or AD alone. In the present included studies, some publications included purely endometriosis-associated infertility or purely adenomyosis-associated infertility in the study group. Among the other studies, adjustments were made for patients with other factors of infertility between the two groups. Therefore, the robustness of the present finding was proven, indicating that women who suffer from EMS in SC or AD during ART should be included among those who may need closer prenatal monitoring and follow-up to prevent miscarriage.

The present study demonstrated that compared with women with tubal infertility during ART, women with EMS I/II or EMS III/IV had a similar miscarriage risk, respectively. However, one included study revealed that women with EMS I/II or EMS III/IV had a higher miscarriage risk in SC, when compared with those without EMS, separately. It was reported that there was no obvious difference observed in miscarriage risk when 238 women with EMS III/IV were compared with 439 women with stage I/II EMS during ART [52]. At the same time, a similar miscarriage risk was observed between 674 women with stage III/IV EMS and 681 women with EMS I/II. In addition, the early and late stages of EMS were observed to share similar epidemiological characteristics, suggesting an epidemiological (and pathogenetic) continuum between different stages of EMS [53]. The present results imply that with the increase in staging of EMS, miscarriage risk appeared not to show significant differences. In the present included papers, unresected and resected OMA were both not found to be associated with miscarriage. At the same time, the surgical and expectant management of OMA in infertile women prior to ART did not show significant differences in miscarriage risk, suggesting that OMA might not be the main causative factor of spontaneous abortion [54]. Therefore, there might be a lack of sufficient evidence to remove OMA before pregnancy. It is recommended to adopt a conservative treatment plan in the long-term management of OMA. Furthermore, it was revealed that DIE was associated with miscarriage and that women with SUP had a higher miscarriage risk. However, the surgical excision of the DIE did not significantly decrease the incidence of miscarriage [55, 56]. In addition, in the following laparoscopic surgery for SUP, the diminished ovarian reserve resulted in the adverse prognosis for pregnancy [57]. Considering the lack of number of studies and sample size, the observation should be cautiously interpreted. Larger high-quality studies are expected to verify these present results in the future.

A systematic review considered that in the second half of pregnancy, the EMS appeared not to have negative effects on pregnancy outcomes [58]. In the present study, compared with those without EMS, women with EMS had a higher early abortion risk, while late abortion risk was similar in women with or without EMS. In addition, women with EMS had a higher early abortion risk (at <12weeks) than late abortion risk (at ≥12 weeks). It was revealed that women with EMS appeared to be associated with first-trimester spontaneous abortion [59]. The limited data available for analysis should be highlighted. Future studies are required to determine whether women with EMS are more likely to have early pregnancy loss.

The pathophysiology of EMS and AD remains poorly understood. However, growing studies have suggested that oxidative stress, inflammation factors/cytokines, angiogenesis, and hormonal interactions play major roles in EMS [60–63]. Meanwhile, sex hormone receptors, junctional zone disruption, and inflammatory factors are considered the causal factors for AD [64, 65]. It has been reported that an increased expression level of nitric oxide species (eNOS) and reactive oxygen species (ROS) in oxidative stress can influence the oocyte and embryo quality, which leads to declined embryo implantation rate in EMS patients [61]. It was reported that attenuated progesterone action might be the basis for the implantation failure in EMS [66]. Vascularization was considered a major pathogenesis in EMS. Proper endometrial vascular development was considered crucial for successful embryo implantation. However, abnormal angiogenesis and uterine natural killer cell (uNK cell) number/function might result in reproductive failure [64]. Disturbances in vascular development might be a causal factor in spontaneous abortion. In addition, it was reported that an increased number of CD56+ uNK cells were detected in the peri-implantation endometrium from women with recurrent miscarriage [67]. It was interesting that the EMS and AD frequently coexisted [68, 69]. The presence of oxidative stress and anomalies in free-radical metabolism might alter the uterine receptivity in EMS and AD. The abnormal endometrial milieu and endometrial dysfunction in EMS and AD contributed to the adverse pregnancy outcome through hormonal, metabolic, and inflammatory mechanisms [70]. Among these theories, inflammatory mechanisms were considered more relevant in EMS and AD. Overall, further researches are required to confirm the biochemical links between EMS and AD and miscarriage to develop preventive measures.

The present study had several strengths. A large amount of studies had allowed for the subgroup analyses to prove the robustness of the results, and subgroup analyses were carried out to evaluate the miscarriage risk by week of pregnancy loss, which has not yet been reported in prior literatures [71]. In addition to reporting the miscarriage risk in women with EMS, the investigators also reported some important reproductive outcomes that were not presented in previous reviews [50], such as ectopic pregnancy. The limitations of the present study were affected by the quality of each of the included studies and the heterogeneity of the overall eligible publications. Since the diagnostic methods were not restricted, the diagnoses of EMS or AD were not uniform between studies. The included studies differed in the selection of control groups with the use of fertility women and subfertility women as the controls. One potential limitation was that unpublished studies were not searched, which might limit the comprehensiveness of retrieved literatures. In addition, since the articles that reported positive results were more likely to be published, the present study had a potential risk of reporting bias.

## 5. Conclusions

Women with EMS have an augmented miscarriage risk, when compared to those without EMS in SC, and women with EMS have a similar miscarriage risk, when compared to those with tubal infertility during ART. Meanwhile, it is found that women with EMS have higher odds of early abortion (<12 weeks). Miscarriage risk increases in women with AD using ART. With the increase in staging of EMS, miscarriage risk appears not to show significant differences. Women with SUP and DIE have an increased miscarriage risk, respectively, while unresected and resected OMA are both not observed to be associated with miscarriage. These present findings suggest that pregnant women with EMS in SC or AD during ART may require closer prenatal monitoring and follow-ups to prevent miscarriage, especially in the first trimester (<12 weeks). Furthermore, a consensus on its accurate recording is required in future studies, including the types and stages of EMS and week of miscarriage.

## Figures and Tables

**Table 1:** Characteristics of identified literature.

Authors (year)	Study design	Sample size	Study location	Mode of conception	Type of disease	Exposure ascertainment	Outcomes
Porpora et al. (2020)	Prospective cohort study	425	Italy	SC	EMS	Surgical/clinical/instrumental diagnosis	Miscarriage, PPH, IUGR, gestational diabetes, stillbirth, low birthweight preterm birth, placenta praevia, placental abruption, preeclampsia
Farland et al. (2019)	Prospective cohort study	196722	America	SC	EMS	Laparoscopic diagnosis	Miscarriage, gestational diabetes, ectopic pregnancy, stillbirth, low birthweight, preterm birth
Mekaru et al. (2014)	Retrospective cohort study	108	Japan	SC	EMS	Laparoscopic evaluation	Miscarriage, low birthweight, preterm birth
Pittaway et al. (1988)	Retrospective cohort study	350	America	SC	EMS	Laparoscopy or laparotomy	Miscarriage, ectopic pregnancy
Hjordt Hansen et al. (2014)	Retrospective cohort study	123335	Denmark	SC	EMS	Discharge diagnosis by the international classification of diseases	Miscarriage, ectopic pregnancy
Santulli et al. (2016)	Retrospective cohort study	1851	France	SC	EMS	Surgical examination of the abdominopelvic cavity	Miscarriage
Saraswat et al. (2016)	Retrospective cohort study	13665	Scotland	SC	EMS	Laparoscopy or laparotomy	Miscarriage, PPH, APH, low birthweight, stillbirth, ectopic pregnancy, preterm birth, placenta praevia, placental abruption
Geber et al. (1995)	Retrospective cohort study	1506	London	IVF	EMS	Laparoscopy	Miscarriage, ectopic pregnancy
Omland et al. (2005)	Retrospective cohort study	1026	Norway	IVF/ICSI	EMS	Laparoscopic diagnosis	Miscarriage, ectopic pregnancy, stillbirth
Kuroda et al. (2009)	Case control study	82	Japan	IVF/ICSI	EMS	Laparoscopic surgery/ultrasound/MRI	Miscarriage
Olivennes et al. (1995)	Retrospective cohort study	325	America	IVF	EMS	Laparoscopic evaluation	Miscarriage
Polat et al. (2014)	Retrospective cohort study	616	Turkey	IVF	EMS	Laparoscopy or laparotomy, transvaginal ultrasonography	Miscarriage
Guo et al. (2016)	Retrospective cohort study	437	China	IVF	EMS	Laparoscopy or laparotomy	Miscarriage
Pop et al. (2014)	Prospective cohort study	235	Serbia	IVF	EMS	Surgically confirmed	Miscarriage
Sharma et al. (2020)	Prospective cohort study	652	India	IVF	EMS	Laparoscopic diagnosis	Miscarriage, PPH, APH, gestational diabetes, preterm birth, IUGR, preeclampsia
Matalliotakis et al. (2007)	Case control study	174	Greece	IVF-ET	EMS	Laparoscopic diagnosis	Miscarriage
Curtis et al. (1993)	Retrospective cohort study	206	England	IVF-ET	EMS	Laparoscopic diagnosis	Miscarriage, ectopic pregnancy
Arici et al. (1996)	Case control study	105	America	IVF-ET	EMS	Laparoscopic diagnosis	Miscarriage
Bergendal et al. (1998)	Retrospective cohort study	146	Canada	IVF-ET	EMS	Laparoscopic diagnosis	Miscarriage, ectopic pregnancy
Pabuccu et al. (2004)	Randomized controlled trials	171	Turkey	ICSI	EMS	Laparoscopic diagnosis	Miscarriage
Mathieud et al. (2010)	Retrospective cohort study	526	France	IVF	EMS	Sonography, MRI	Miscarriage
Kim et al. (2011)	Prospective cohort study	40	Korea	IVF-ET	EMS	Laparoscopic diagnosis	Miscarriage
Kuivasaari et al. (2005)	Retrospective cohort study	185	Finland	IVF/ICSI	EMS	Laparoscopic diagnosis	Miscarriage, ectopic pregnancy
Opoien et al. (2012)	Retrospective cohort study	2245	Norway	ICSI	EMS	Laparoscopic diagnosis	Miscarriage
Singh et al. (2013)	Case control study	340	India	IVF	EMS	Laparoscopic diagnosis	Miscarriage
Senepati et al. (2016)	Retrospective cohort study	347185	Washington	IVF	EMS	Laparoscopic diagnosis	Miscarriage
Vaz et al. (2017)	Retrospective cohort study	181	Brazil	IVF	EMS	Laparoscopy or MRI	Miscarriage
Esinler et al. (2006)	Case control study	156	Turkey	IVF/ICSI	EMS	Laparoscopic diagnosis	Miscarriage
Bahceci et al. (2005)	Retrospective cohort study	1244	Turkey	ICSI	EMS	Laparoscopic diagnosis	Miscarriage
Sharma et al. (2019)	Retrospective cohort study	973	India	IVF	EMS and AD	EMS confirmed by laparoscopy, AD diagnosed by TVS	Miscarriage, PPH, APH, preterm birth, IUGR, preeclampsia
Costello et al. (2011)	Retrospective cohort study	201	Australia	IVF/ICSI	AD	Transvaginal ultrasound	Miscarriage
Youm et al. (2011)	Case control study	154	Korea	IVF-ET	AD	TVS	Miscarriage
Thailluri et al. (2012)	Retrospective cohort study	213	Australia	IVF-ET	AD	TVS	Miscarriage
Benaglia et al. (2014)	Case control study	98	Italy	IVF/ICSI	AD	TVS	Miscarriage
Hashimoto et al. (2017)	Case control study	294	Japan	ART	AD	MRI/TVS	Miscarriage
Martinez-Conejero et al. (2011)	Retrospective cohort study	443	Spain	ART	AD	TVS	Miscarriage
Yan et al. (2014)	Retrospective cohort study	154	China	ART	AD	Transvaginal ultrasound	Miscarriage, ectopic pregnancy
Salim et al. (2012)	Prospective cohort study	275	London	ART	AD	Transvaginal ultrasound	Miscarriage
Schwartz et al. (2017)	Cross-sectional study	940	Switzerland	SC or ART	EMS	Surgical diagnosis	Miscarriage

SC: spontaneous conception; ART: assisted reproductive technology; IVF: in vitro fertilization; ICSI: intracytoplasmic sperm injection; EMS: endometriosis; AD: adenomyosis; TVS: transvaginal sonography; MRI: magnetic resonance imaging; APH: antepartum hemorrhage; PPH: postpartum hemorrhage; IUGR: intrauterine growth restriction.

**Table 2:** Newcastle-Ottawa risk of bias for included studies.

Authors (year)	Selection of study group score	Comparability of group score	Ascertainment of exposure or outcome score	Total NOS score	Risk of bias (low, medium, high)
*Retrospective cohort study*					
Omland et al. (2005)	3	2	2	7	Low
Martinez-Conejero et al. (2011)	3	2	2	7	Low
Hjordt Hansen et al. (2014)	3	1	3	7	Low
Yan et al. (2014)	3	2	2	7	Low
Santulli et al. (2016)	3	2	3	8	Low
Saraswat et al. (2016)	3	2	3	8	Low
Sharma et al. (2019)	3	1	3	7	Low
Pittaway et al. (1988)	3	1	3	7	Low
Geber et al. (1995)	4	2	2	8	Low
Olivennes et al. (1995)	3	2	2	7	Low
Mekaru et al. (2014)	3	2	2	7	Low
Polat et al. (2014)	3	2	3	8	Low
Guo et al. (2016)	3	2	2	7	Low
Costello et al. (2011)	3	2	2	7	Low
Mathieud et al. (2010)	3	2	2	7	Low
Senepati et al. (2016)	3	1	2	6	Medium
Curtis et al. (1993)	3	2	1	6	Medium
Bergendal et al. (1998)	3	2	2	7	Low
Kuivasaari et al. (2005)	3	2	2	7	Low
Opoien et al. (2012)	3	2	2	7	Low
Vaz et al. (2017)	3	1	2	6	Medium
Bahceci et al. (2005)	3	0	3	6	Medium
Thailluri et al. (2012)	3	2	2	7	Low
*Prospective cohort study*					
Pop et al. (2014)	3	2	2	7	Low
Kim et al. (2011)	3	2	3	8	Low
Salim et al. (2012)	3	2	2	7	Low
Farland et al. (2019)	3	1	3	7	Low
Porpora et al. (2020)	4	1	2	7	Low
Sharma et al. (2020)	3	2	2	7	Low
*Case control study*					
Arici et al. (1996)	3	1	2	6	Medium
Singh et al. (2013)	3	2	2	7	Low
Kuroda et al. (2009)	3	1	2	6	Medium
Esinler et al. (2006)	3	2	2	7	Low
Benaglia et al. (2014)	3	2	2	7	Low
Matalliotakis et al. (2007)	3	2	2	7	Low
Hashimoto et al. (2017)	3	2	2	7	Low
Youm et al. (2011)	4	0	2	6	Medium
*Cross-sectional study*					
Schwartz et al. (2017)	3	2	2	7	Low

**Table 3:** Risk of bias for randomized controlled trials.

Bias	Selection		Performance	Attrition	Reporting	Other sources of bias
Studies (year)	Random sequence generation	Allocation concealment	Blinding	Incomplete outcome data	Selective reporting	
Pabuccu et al. (2004)	Low risk	Unclear	Unclear	Low risk	Low risk	Low risk

## Data Availability

The data used to support the findings of this study are included within the article.
